# Considerations when introducing electronic patient-reported outcome data capture in multicentre oncology randomised controlled trials

**DOI:** 10.1186/s13063-022-06955-w

**Published:** 2022-12-12

**Authors:** Lara Philipps, Stephanie Foster, Deborah Gardiner, Alexa Gillman, Joanne Haviland, Elizabeth Hill, Georgina Manning, Morgaine Stiles, Emma Hall, Rebecca Lewis

**Affiliations:** Clinical Trials and Statistics Unit, The Institute of Cancer Research, London, SM2 5NG UK

## Background

The Cancer Research UK-funded Clinical Trials and Statistics Unit at The Institute of Cancer Research (ICR-CTSU) is an academic clinical trials unit which designs, conducts, and analyses investigator-initiated, non-commercial multicentre oncology trials. Trials are conducted in the secondary care setting at hospitals in the UK’s National Health Service and internationally. The ICR-CTSU portfolio includes clinical trials of investigational medicinal products (CTIMPs) and non-CTIMPs investigating radiotherapy and surgery. Key disease areas of interest include breast, prostate, bladder, lung, and head and neck cancers.

Assessment of patient-reported outcomes (PRO) is a key secondary endpoint for many ICR-CTSU trials and its use as a primary endpoint is increasing. PRO are collected via validated questionnaires completed on paper by trial participants. The questionnaires capture the impact that treatment and health conditions may be having upon their symptoms and quality of life—defined as “any report of the status of a patient’s health condition that comes directly from the patient, without interpretation of the patient’s response by a clinician or anyone else” [1]. Questionnaires are either administered to participants by research teams in their local hospitals or sent to patients’ home addresses by ICR-CTSU, after confirmation from local hospital site staff that the participant is alive and able to complete the booklet.

In 2012, the ICR-CTSU replaced use of paper case report forms with electronic data capture (EDC) directly from participating sites and this is now the primary method of clinical data collection. Over the past decade, advances in information technology and improved access to the Internet have led to a rapid increase in the use of electronic devices including smartphones, tablets, and laptops across the UK population. In 2020, 92% of adults in the UK regularly used the internet [2], with usage by the over 75 s increasing from 29% in 2013 to 54% in 2020. The effect of the COVID-19 pandemic is likely to have increased internet exposure further and it has already been shown that the proportion of adults online aged over 65 who made at least one video-call each week increased from 22% in February 2020 to 61% by May 2020 [3].

Given the increasing use of the internet and electronic devices by the UK population, following our successful roll out of electronic capture of clinical data, we would like to offer our trial participants the option of using an electronic system to complete PRO questionnaires.

Outside clinical trials, there has been extensive work across different medical specialities to establish intra-patient equivalence of paper and electronic PRO questionnaires and their validity for data collection. Muehlhausen et al. [4] conducted a systematic review and meta-analysis of the equivalence of patient-reported outcome measures administered using electronic and paper formats. The review included 72 intra-patient studies, showing overall equivalence between the two formats when completed by the same patients. This was an update of a previous review by Gwaltney et al. conducted in 2008 [5], which also showed equivalence within the same patients. Following these two meta-analyses, further studies have added weight to the finding of within-patient equivalence of data following migration from paper to electronic format. Participants of these studies had the equivalence of scores compared between their completion of both paper and electronic questionnaires, with a paper test–retest arm as the control [6, 7].

Trial participants’ willingness to complete ePROs would be essential to maximise the completeness of data returned to assess PRO endpoints. One factor which may increase questionnaire return rates and improve the patient experience with ePROs in comparison to paper is a reduction in the amount of time required to complete questionnaires. Park et al. showed that the time taken to complete an electronic questionnaire in a clinical outpatient setting was significantly shorter than that required for the paper version [8]. Some studies have shown 83% compliance with ePROs in the clinic, with between 76 and 95% of patients finding a system usable and recommending it to others [9, 10].

There are two significant limitations in the literature published to date. Firstly, although ePROs are becoming increasingly popular for use in clinical trials, to our knowledge there is limited published evidence of patient uptake and compliance in this setting in comparison to paper completion, and none from randomised studies of the mode of PRO completion. One study including rheumatoid arthritis patients within two randomised controlled trials asked participants to complete electronic diaries. This study showed high compliance of up to 93% of patients over 12 weeks; however, there was no control group completing paper questionnaires, meaning it is not possible to be sure whether the compliance was non-inferior to paper diary use [11]. Clinical trials in a surgical setting found poor uptake of ePROs amongst participants offered the choice. In a report of two trials from 2019, only 12% of 642 participants opted for completion of ePRO in one study and 34% of 1296 participants opted for it in another. Overall, 280 of 5700 expected questionnaires were completed electronically (5%), with the remainder completed on paper [12]. It is likely that there would be population-level differences between trial participants who choose to complete paper questionnaires and those who would prefer to complete electronic questionnaires which may impact the responses given; however, to our knowledge, there are no published data exploring this.

The second limitation is the lack of information about whether the completeness of data is equivalent or superior in the electronic format. At the ICR-CTSU, we have extremely high return rates of paper PRO questionnaires, particularly at early timepoints. On review of 10 recent trials, there was a median questionnaire return rate of 76% at the first post-trial intervention time point. In trials where the PRO was the primary endpoint, this increased to > 90%.

One recent study in a healthy university undergraduate population [13] assessed data capture using electronic and paper, with participants of a prospective study being given the opportunity to choose the format for completion of food intake questionnaires at baseline and 10-year follow-up. The results were mixed, with increased missing data in some subsections in the electronic version and improved data levels in other subsections. The study concluded that the number of questions correctly filled in was equivalent between electronic and paper questionnaires. However, these results may not be applicable in a patient population, particularly in oncology where patients can be unwell and are more likely to be an older cohort [14]. There is a limited number of other studies but these again are largely in either the mental health, general healthy, or paediatric populations [15, 16] and therefore not directly applicable to an oncology patient population. It should also be taken into consideration that there could be a difference in responses to paper or electronic questionnaires, for example participants with worse QoL may find an electronic questionnaire easier since the need to return it to a post box is removed, or alternatively those selecting paper questionnaires may be more likely to be older and frailer and have a lower overall QoL.

Here we discuss the considerations, potential barriers, and benefits of introducing ePRO data collection within the cancer clinical trial setting.

## Ethical and regulatory requirements

The ethical and regulatory requirements associated with data capture within clinical trials are critical considerations when introducing ePRO. Although the UK’s Medicines and Healthcare products Regulatory Agency (MHRA) are yet to issue specific guidance with respect to ePROs, there have been critical findings in recent MHRA GCP inspections [17, 18]. The GCP inspections metric report of 2018–2019 reported that “There was incorrect data in the eDiary that could not be changed, but was used for the analysis” and that “The eDiary devices used by subjects did not have an audit trail.” Similarly, in the 2017–2018 MHRA GCP inspections metric report there was concern about insufficient documentation of user acceptance testing for electronic patient diaries.

Outside the UK the FDA guidance for industry: Electronic Source Data in Clinical Investigations published in 2013 [19] noted that the subject of the PRO when electronic should be listed as the originator and the eCRF should be the source. Further guidance has not yet been published. The European Medicines Agency (EMA) published a “Reflection paper on expectations for electronic source data and data transcribed to electronic data collection tools in clinical trials” [20]. This highlighted the importance of an instrument being “an accurate representation of the protocol ensuring that the data … can be captured correctly and that the …subject response is not biased by default values present within the instrument” and that “The clarification process for data entered by trial subjects should be documented and it should be clearly stated where changes to data entered by subjects will not be made.”

Further ethical challenges need to be considered in the use of ePROs within oncology trials, particularly those in cancer types occurring more frequently in older patient populations. Although internet use is becoming increasingly widespread, as discussed above, there remains a substantial proportion of over 75 s who do not regularly use the internet. Although we wish to offer our trial participants the option to complete questionnaires online, we do not wish to exclude people from contributing to our PRO studies for lack of internet access or disinclination to complete questionnaires electronically.

We therefore are planning to implement ePRO in parallel to our existing paper PRO collection systems in order that we do not disenfranchise any of our trial participants. This adds an additional layer of complexity to the use of ePRO, as the approach needs to mirror that used for paper collection to avoid introduction of systematic bias between modalities, but we believe this is crucial to ensure that possibly underrepresented digitally marginalised groups can continue to contribute their experience.

## Introduction and assessment of ePRO data collection

**Fig. 1:**
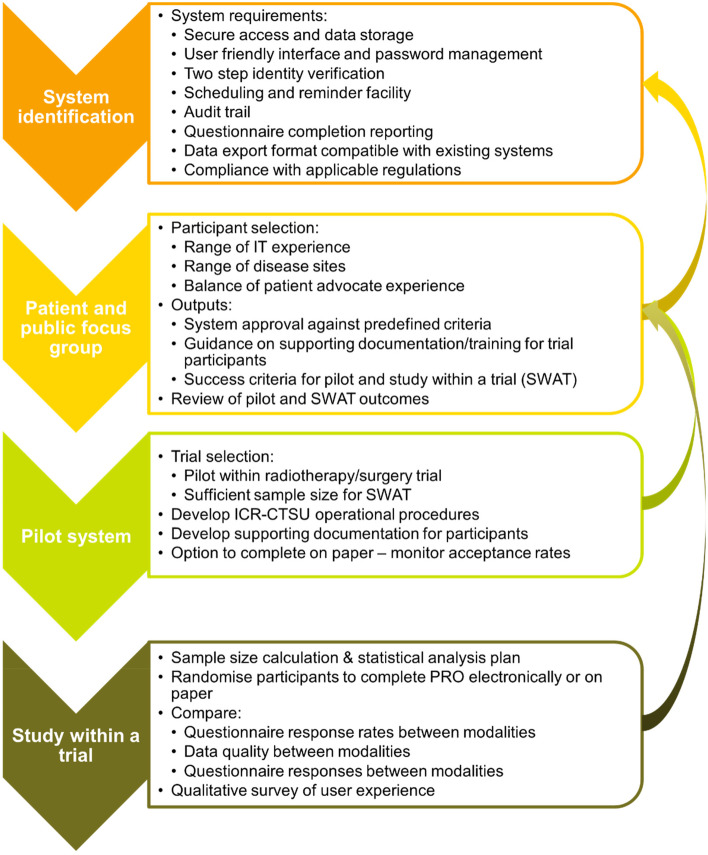
Key steps for the introduction of ePRO and considerations required for each step

We have developed a plan to systematically introduce and assess use of ePROs in ICR-CTSU trials, including robust assessment of potential systems, integral patient and public involvement, and development of a pilot study to assess acceptability of ePRO to our trial participants and impact of questionnaire format on data reported. Figure 1 outlines our key considerations for each step.

## System identification and setup

We developed a full system requirements specification to help review and assess potential systems for ePRO introduction (Table 1). The system is required to have a user-friendly interface with an identification verification system and password management. Secure access, data storage, and usability of data are paramount, and it must comply with all applicable regulations. The system is also required to manage ePRO distribution and oversight including automatically sending emails to participants when questionnaires are due, reminding them when these have not been returned, and providing reporting functionality for central study oversight, to mirror existing paper questionnaire management systems.

**Table 1:** ePRO system requirements and acceptance criteria

ePRO system requirements and acceptability criteria
Participant experience
User-friendly and acceptable to trial participants
Compatible with participants’ own devices and a range of internet browsers
Permits 24/7 questionnaire access
Regulatory, governance, and operational requirements
Meets institutional security, data protection, and system integration requirements
Securely holds data in accordance with good clinical practice (GCP) and general data protection regulatory (GDPR) requirements
Systems and GCP validation documentation
Business continuity, disaster recovery, and back-up capabilities
Affordability for non-commercial trials settings
IT and data management functionality
Ability to program validated PRO instruments and bespoke questionnaires
Adaptability to ensure trial specific questionnaires are available for completion by the participants via a secure internet site and/or a mobile application according to host trial protocol specified timing
Allows questionnaires to be submitted only once and removes previously submitted questionnaire responses from participants’ view
Provides automatic electronic reminders to participants when questionnaires are due to be completed
Removes access to uncompleted questionnaires after a specified time period has elapsed
Provides a scheduling facility to mirror protocol requirements and existing paper questionnaire management systems • Allows clinical trial unit staff to view the participants’ questionnaire schedules • Allows clinical trial unit staff to set the questionnaire due dates and reminder dates • Allows disablement of future reminders and questionnaires if applicable, e.g. upon withdrawal from study • Allows for participants following different questionnaire schedules within the same trial in accordance with protocol
Capacity to provide validated reports for central study management to mirror existing paper questionnaire management systems, including: • Overdue questionnaires (not started) • Incomplete questionnaires (started but not submitted/completed) • Submitted/completed questionnaires
Compatibility with existing clinical trials unit trial entry and data management systems
Statistical functionality
Provides data in a compatible format for statistical analysis programs

The system used by ICR-CTSU for EDC did not have ePRO functionality; therefore, an alternative needed to be sought. Finding ePRO systems meeting the requirements which are also affordable to an academic trials unit has been challenging, with 17 systems having been assessed. A system was identified that would fulfil the system requirements outlined above and following this significant time was required for system setup. This included converting validated QoL questionnaires used within our clinical trials into electronic versions which mirror the existing paper questionnaire formats. This was completed with the agreement of the licence holders of the questionnaires as no current validated electronic formats exist. Recommendations from International Society for Pharmacodynamics and Outcomes Research (ISPOR) group and Muehlhausen et al.’s subsequent qualitative synthesis regarding migration were followed [21] and the format of the questionnaire was minimally altered from paper to electronic versions.

Due to the non-commercial trial setting, it was not possible to fund the purchase of tablets for patients to use in clinic, so consideration was taken to account for the different technology that participants may own, including mobile phones, tablets, and laptop computers. The decision was taken to use a system that sent participants an email link to complete questionnaires to allow all technology to be used, rather than mobile applications which would likely be limited to specific platforms.

The set-up of this system required significant information technology support from within the clinical trials unit. It was recognised that no system was without flaws; however, with the funding available to an academic institution, time was spent to design the system to the best possible impact.

## Patient and public involvement

Patient and public involvement (PPI) in the design of health research is integral to ICR-CTSU’s work and can enhance enrolment and retention by improving trial design, recruitment and retention strategies, and patient-facing material [22]. As we are implementing a new system for use by our trial participants, PPI is an integral part of this project.

Firstly, we developed a public survey investigating attitudes to completing health questionnaires online and capturing views on introducing ePROs. A key aim was to reach people with diverse demographics and a range of IT experience to obtain as broad and representative review of attitudes to ePRO as possible; however, unfortunately, this aim was somewhat hampered as the survey was conducted during the pandemic. To try and reach a less IT literate population, the survey was advertised via newspaper adverts, sent directly to patients after a telephone clinic appointment, and advertised in online patient forums.

In total, 13/50 (26%) completed the survey on paper and 37/50 (74%) online. 47/50 (94%) had regular access to the internet either at home or on their mobile telephone. 38/50 (76%) of respondents would rather fill out a health questionnaire online and of those who would rather fill it out on paper 6/11 (54.5%) would be happy to complete online if requested. The participants in the survey who did not have access to the internet were all aged 71 or older and were in the lowest educational and income bracket, underlining the requirement to maintain the option of completing patient-reported outcomes on paper in clinical trials, to prevent the systematic exclusion of trial participants in under-served groups.

Survey respondents were asked if they would be interested in joining a focus group on the planned ePRO pilot study design and eight survey participants took part. Recommendations from these groups were subsequently included in the study protocol, which will be published separately to this commentary.

All participants of the focus groups also joined the ePRO pilot study patient and public oversight committee to assist with ongoing study management. This has been essential in the continued set-up of the ePRO system, with six group members testing the online completion of ePRO using their personal devices, providing feedback on the usability and acceptability of the system which was incorporated prior to launch.

## Study to pilot ePRO introduction

A randomised study has been designed with the support of our patient and public collaborators. This study within a trial (SWAT) will pilot and assess the introduction of ePRO within ICR-CTSU trials and obtain evidence to fill some of the gaps in the literature described above. It will sit across multiple ICR-CTSU host trials to pragmatically assess ePRO uptake, completion rates, and impact on data reported across different patient populations within a randomised controlled trial, rather than a theoretical setting. The study will be a partially randomised patient preference trial which will allow trial participants the option to choose to fill out the PRO questionnaire either on paper or electronically if they have a strong preference, preventing the inadvertent exclusion of participants and potential damage to QoL data capture rates within the host trials. Further details of the SWAT will be published in a separate protocol paper.

The long-term strategy will be to review the results of the pilot study and, with continued PPI input, routinely implement the use of ePROs in ICR-CTSU clinical trials should the evidence be supportive.

## Conclusion

Identification of an affordable, user-friendly, adaptable, and compatible ePRO system has proved to be a complex process requiring the review of multiple systems and involvement of multiple internal and external stakeholders. The resource required to implement ePRO data capture to ensure its long-term reliability and acceptability to our trial participants is substantial and requires a multi-disciplinary approach. The ICR-CTSU is fortunate to have a dedicated team of clinical trial IT and data management professionals embedded within the unit, as well as PPI and trial conduct methodology expertise to allow detailed consideration of the requirements of ePRO introduction and assessment of impact within a randomised setting.

Use of ePRO even in a world of increasing technology use is a complex and multifactorial project requiring careful consideration and adequate resourcing to ensure that both end users and clinical trial units benefit from its introduction and no trial participants are inadvertently disenfranchised.

## Data Availability

Not applicable
